# Cases and Remarks

**Published:** 1827-08

**Authors:** T. Chevalier


### SMALL-POX AFTER VACCINATION.

*Cases and Remarks by the late* T. Chevalier, Esq. F.R.S. F.A.S. F.L.S. &c.

C. H. T—, Esq. an intimate friend of mine, and an architect of distinguished eminence, has a family of nine children, all of whom were vaccinated by me in their infancy. My friend himself had suffered severely from the small-pox in his early age, so as to leave ground for an apprehension that the constitutions of his children might be predisposed to its malignancy, if it should happen to invade them.

On the 7th of October, 1822, Julia, aged eleven years, was walking out with her mother, when a child passed them who had the small-pox. Her mother did not notice the circumstance, but the young lady herself immediately mentioned it in a very marked manner, and she afterwards told me that she felt at the instant a peculiar shuddering. On the 14th, she was attacked with the symptoms which usually precede the variolous eruption; and on the 16th I was requested to visit her. I found her with an efflorescence of the skin, which I did not then believe to be small-pox. There was, however, one point on the right cheek which looked like it; and on the 18th the disease was too well characterised to admit of any doubt as to its nature, though of the mildest character; the efflorescence of the skin (which, in the uncontrolled state of the disease, I have always observed to prognosticate its most malignant form,) having entirely disappeared.

On the 20th, the eruption appeared perfect, but the pustules small; and from that time it declined, without any thing like secondary fever. On the 28th, there was slight inflammation and swelling of the right upper eyelid, sufficient to irritate the surface of the eye; but it soon yielded to mild treatment, and she has since continued in perfect health.

The disease, however, having thus taken place in the family, naturally excited anxiety about the rest of the children, in whom the consequences appeared as follows:

Edmund, the youngest, aged seven months, who had been successfully vaccinated by two punctures in each arm, and who was not withdrawn from the nursery during the illness of his sisters, had no symptom of the disease whatever.

Georgiana, aged two years, had been vaccinated in one point in each arm. She had a little feverishness for two nights, which commenced on the 4th of November, but was followed by no eruption, and would in all probability have excited no suspicion of small-pox, if it had not already pre-existed in the family.

Maria, aged six years, was attacked with the constitutional symptoms distinctly on the 2d of November. Only one spot appeared, which was on her upper lip; and she was perfectly well on the 9th.

Augusta, aged seven years, began to sicken on the 4th. The eruption appeared on the 7th, but it was very slight; and, though she had rather more debility than the rest during its course, it faded away in an equally short time.

Harriet, aged nine years, was attacked on the 3d. On the 7th, a very slight eruption appeared, the characters of which were scarcely recognisable; and on the 9th, she was perfectly well.

Arthur, aged fourteen, had some symptoms which, being similar to those of an attack of small-pox, excited an expectation that he also would have the disease; but he was well after two days, without any eruption.

Frederick, aged seventeen, was attacked rather severely on the 2d, with considerable fever. The eruption manifested itself on the following day; it was of the mildest nature. On the 7th, it began to fade, when he felt perfectly relieved, and was well in a few days.

Caroline, the eldest, had been vaccinated in both arms in her infancy, by one puncture in each, and had suffered from considerable inflammation in both of them during the progress of her inoculation: she, however, escaped every symptom of the small-pox.

I have not the smallest doubt that, if vaccination had not preceded the infection in these cases, the consequence to this interesting family would have been melancholy in the extreme. Perhaps, no series of facts in the history of vaccination, so closely connected, can better exemplify the gradations of the beneficial influence of this invaluable preventive, or more clearly show the propriety of the recommendation formerly promulgated by the Board, to inoculate the vaccine matter into at least two places in each arm, in order most fully to secure its salutary effects upon the constitution by an adequate degree of the specific inflammation. It also demonstrates the propriety of absolutely prohibiting, by legislative authority, the exposure of those objects in the public roads and streets, who through ignorance, or by accident or error, may have become the subjects of the destructive and loathsome influence of a disease, by which, according to the Bills of Mortality, a fifth part of the population of this metropolis was formerly destroyed.*

I wish to add to this account, that, having been recently engaged in some rather extensive investigations concerning the structure of the skin, I find that the excretory ducts (of which there are very many millions on the surface of the body, serving different and important offices,) are for the most part obliterated in the foveæ, or pits, left by severe small-pox. So that a most serious change must suddenly and unavoidably occur in some of the most important functions in the animal economy; especially when the disease occurs, as it usually does, in the progress of growth, when nothing can be more essential than the integrity of the circulation of the blood on the surface of the body, and the perfection of those operations which are dependent thereupon. Hence we may account, in a great degree, for the excitement of those diseases of glands, of the eyes and eyelids, of the bones, and of other organs, which small-pox is so frequently seen to induce; and, although the balance may in time be restored in many instances, by the gradually increased determination to so much of the skin as remains uninjured, yet the alteration, so long as it operates, must be both injurious and perilous.

A remedy, therefore, like vaccination, which, in almost every case, if not in every case in which it has been properly performed, prevents the secondary fever, the ulceration, or gangrene, and the consequent pits, from occurring in small-pox, (even if it fail to exempt the constitution altogether from the

* It is still questionable whether the *small-pox* does not *recur a second time*, in quite as large a proportion of cases as it is found to follow a regular vaccination; in which case the latter is quite as effectual a security against the variolous infection as small-pox itself.—T. C.

disease,) although it had no other advantage, would be of incalculable value to society, and indisputably of the highest advantage to every individual upon whom it is bestowed.

*20, South Audley-street*; — 1822.

